# Physical activity, patient-reported symptoms, and clinical events: Insights into postprocedural recovery from personal digital devices

**DOI:** 10.1016/j.cvdhj.2021.06.002

**Published:** 2021-07-03

**Authors:** Victoria L. Bartlett, Joseph S. Ross, Nilay D. Shah, Laura Ciaccio, Joseph G. Akar, Peter A. Noseworthy, Sanket S. Dhruva

**Affiliations:** ∗ Yale School of Medicine, New Haven, Connecticut; † Section of General Internal Medicine and National Clinician Scholars Program, Yale School of Medicine, New Haven, Connecticut; ‡ Department of Health Policy and Management, Yale University School of Public Health, New Haven, Connecticut; § Center for Outcomes Research and Evaluation, Yale–New Haven Hospital, New Haven, Connecticut; ¶ Division of Health Care Delivery Research, Kern Center for the Science of Health Care Delivery, Mayo Clinic, Rochester, Minnesota; ‖ Department of Internal Medicine, Cardiovascular Medicine, Yale School of Medicine, New Haven, Connecticut; ∗∗ Department of Cardiovascular Medicine, Mayo Clinic, Rochester, Minnesota; †† Section of Cardiology, Department of Medicine, University of California–San Francisco School of Medicine, San Francisco, California; ‡‡ San Francisco Veterans Affairs Health Care System, San Francisco, California

**Keywords:** Ablation, Activity, Atrial fibrillation, Bariatric surgery, Digital health, Patient-reported outcomes, Postprocedural recovery, Remote monitoring, Wearable devices

## Abstract

**Background:**

Personal digital devices may offer insights into patient recovery and an approach for remote monitoring after procedures.

**Objective:**

To examine associations between activity measured using personal digital devices, patient-reported outcome measures (PROMs), and clinical events among patients after catheter ablation for atrial fibrillation (AF) or bariatric surgery.

**Methods:**

We aggregated personal digital device, PROM, and electronic health record data in a study conducted at 2 health systems. We used Fitbit devices for step count assessments, KardiaMobile for cardiac rhythm assessments, and PROMs for pain and palpitations over 5 weeks.

**Results:**

Among 59 patients, 30 underwent AF ablation and 29 bariatric surgery. Thirty-six patients (63%) reported pain. There was no difference in median [interquartile range] daily steps between patients with and those without pain (4419 [3286–7041] vs 3498 [2609–5888]; *P* = .23). Among AF ablation patients, 21 (70%) reported palpitations. Median daily steps were lower among those with palpitations than among those without (4668 [3021–6116] vs 8040 [6853–10,394]; *P* = .03). When accounting for within-subject correlation, recordings of AF were associated with a significant mean decrease in median daily steps (–351; 95% confidence interval –524 to –177; *P* <.01). Patients who received a new antiarrhythmic drug prescription had AF recorded in a median of 5 [5–5] of 5 total weeks, whereas patients who did not receive a new antiarrhythmic recorded AF in a median of 1 [0–3] week (*P* = .02).

**Conclusion:**

Personal digital device and PROM data can provide insight into postprocedural recovery outside of usual clinical settings and may inform follow-up and clinical decision-making. (ClinicalTrials.gov Identifier: NCT03436082)

**Key Findings**

• Among a cohort of patients who underwent atrial fibrillation (AF) ablation or bariatric surgery, there was no significant difference in median daily steps between patients with and those without pain during the first 5 weeks postprocedure.
• Median daily steps were significantly lower among patients who underwent AF ablation and had post-procedural palpitations compared to those without palpitations.
• Among patients who performed at least 1 usable mobile electrocardiographic recording, each AF episode during the week was associated with a significant mean reduction of 351 (95% confidence interval –524 to –177; *P* <.01) median daily steps during that week.
• Leveraging data from patient-reported outcome measures and personal digital devices can inform understanding of patient recovery after procedures, thereby informing follow-up and clinical decision-making.

## Introduction

Personal digital devices, such as Apple Watch® (Apple Inc, Cupertino, CA), Fitbit® (Fitbit, Inc, San Francisco, CA), digital blood pressure cuffs, and mobile electrocardiographic (ECG) devices (eg, KardiaMobile™, AliveCor, Inc, Mountain View, CA), represent novel data sources that can offer insight into patient experiences outside of clinical settings. These devices have been used to characterize physical activity after specific procedures, such as cardiac and orthopedic surgery.^1^, ^2^, ^3^ Lower step counts have been associated with postsurgical complications, increased likelihood of hospital readmission, and increased postoperative symptoms.^4^ However, experience evaluating the association between objective measurements of activity, patient reports of symptoms, and clinical outcomes within the short-term postprocedure period is limited. Analyzing these multiple combined data sources may help to better understand postprocedural recovery and could aid in early detection of clinical deterioration.

Given the growing accessibility of personal digital devices and digital health applications, such analyses are increasingly feasible. Furthermore, novel mobile health (mHealth) technologies can overcome current data siloes by aggregating multiple types of health data from different sources, including patient-generated health data from personal digital devices, patient-reported outcome measure (PROM) data, electronic health record (EHR) data, and pharmacy data.^5^ Because these data can be captured in near continuous fashion, such digital approaches also allow near real-time tracking of postprocedural recovery and, therefore, provide an opportunity to monitor and improve postprocedural patient outcomes.

Atrial fibrillation (AF) ablation and bariatric surgery are 2 increasingly common procedures and thus are well suited for examination of postprocedural recovery enabled by personal digital device data. First, catheter ablation is used for rhythm control of AF, the most common cardiac arrhythmia, which is associated with worse quality of life.^6^^,^^7^ Although early recurrence (within 90 days) of AF is not considered treatment failure, it is associated with long-term recurrence.^8^ Moreover, compared to asymptomatic documented recurrence, early symptomatic AF recurrence has a stronger association with symptomatic long-term recurrence.^9^ Previous studies have characterized early AF recurrence using clinical tools such as Holter monitors, transtelephonic wireless ECGs, implantable cardiac monitors, and, more recently, mobile ECG devices.^10^, ^11^, ^12^, ^13^, ^14^ However, the association of heart rhythms detected by these devices with patient-reported symptoms has not yet been studied. Second, bariatric surgery promotes weight loss and improves metabolic disease and cardiovascular risk factors.^15^ Previous studies that characterized activity, quality of life, and weight loss after bariatric surgery collected data only at specific postprocedure timepoints.^16^, ^17^, ^18^ These studies showed an association between increased physical activity from pre- to post-bariatric surgery with mid- and long-term weight loss.^18^ Short-term postprocedure weight loss also predicts long-term weight loss.^19^^,^^20^ However, no studies have used continuous step count recording during the initial postprocedure period to examine the association of activity with weight lost immediately postprocedure. Using continuously aggregated data from novel personal digital devices provides an opportunity to better understand the associations of patient symptoms and activity in early postprocedural recovery.

We recently demonstrated the feasibility of prospectively aggregating real-world data from multiple sources, including personal digital device, PROM, EHR, and pharmacy data, as part of a cohort study of 60 patients undergoing catheter-based AF ablation or bariatric surgery.^21^ We included 2 different procedures in our study to better understand the generalizability of our research approach in multiple patient populations. However, the content of these data was not evaluated to provide insights into postprocedural recovery. Accordingly, we analyzed these data to study the association of activity with patient-reported symptoms and clinically significant events, including emergency department visits, hospitalizations, and new medication prescriptions. Additionally, among patients receiving AF ablation, we examined the association of activity and symptoms with objective rhythm assessments using a mobile ECG device.

## Methods

### Study design

We conducted an 8-week prospective cohort study of ambulatory patients undergoing ablation for AF or bariatric surgery at 2 academic medical centers, using a novel patient-centered health data sharing platform to aggregate personal digital device, PROM, EHR, and pharmacy (including CVS and Walgreens) data. These methods have been previously described.^21^ For this study, we analyzed data from the first 5 weeks postprocedure, as the pertinent PROM questionnaires did not extend past this point. Patients provided written informed consent and were enrolled in the study before their procedure. This study received institutional review board approval at Yale University and Mayo Clinic. The research reported in this article adhered to the Helsinki Declaration as revised in 2013.

### Step count assessment

All patients were asked to record their activity through step counts using a Fitbit at least once per week. Among patients with at least 1 day of steps recorded during each week, we determined median daily steps for that week. Consistent with previous studies, any days with fewer than 500 steps recorded were considered to have incomplete data for that day and were excluded.^22^^,^^23^ We also determined median daily steps and the proportion of patients who recorded at least 500 steps during at least 1 day in the 5-week study period.

### PROM assessment

Patients were asked about pain (all patients), palpitations (ablation patients only), and appetite (bariatric surgery patients only) through PROM questionnaires twice weekly for the first 5 weeks. We determined the number of patients who completed at least 1 PROM survey. Of these patients, we determined the proportion who reported pain at least once. Among ablation patients, we also determined the proportion who reported palpitations at least once.

### Mobile ECG device reading (AF ablation patients)

Ablation patients were asked to record an ECG using a KardiaMobile (mobile ECG device) at least once per week; patients could perform additional recordings if they wanted. We determined the proportion of AF ablation patients who performed at least 1 mobile ECG recording during the 5 weeks postprocedure. We determined the total number of recordings, the median per patient (including patients who did not perform a recording), and the proportion that was usable (ie, detected as normal sinus rhythm or AF and not as “undetermined,” “too short,” or “no analysis”) during the first 5 weeks postprocedure.

We then examined the content of the recordings, determining the proportion of patients who had at least 1 episode of AF recorded. Among these patients, we determined the median number of total recordings, median number of AF recordings, and median proportion of all usable recordings that were AF.

### Digital scale readings (bariatric surgery patients)

Bariatric surgery patients were asked to weigh themselves using a Withings Body™ (Withings SA, Issy-les-Moulineaux, France) digital weight scale at least once per week; patients could perform additional recordings if they wanted. We determined the proportion of patients who recorded at least 1 weight in both weeks 1 and 5 postprocedure. Among these patients, we determined the percentage body weight lost during that time period and then the median percentage body weight lost across all patients.

### Clinical event (all patients) and antiarrhythmic medications (AF ablation patients) assessment

EHR and pharmacy data, including hospital encounters and medication prescriptions, were aggregated. We identified patients with an emergency department or inpatient encounter during the first 5 weeks postprocedure. Among ablation patients, we identified patients who received a new Class IC or Class III antiarrhythmic drug prescription at least 1 week postablation. We expected prescriptions at discharge, including those filled within the first postprocedure week, to likely be prescribed to prevent AF recurrence.^24^

### Statistical analysis

#### Association between postprocedure week and step count

We conducted a longitudinal analysis of the association of postprocedure week and median daily step count using a generalized estimating equation (GEE) with first-order autoregressive correlation structure. The dependent variable was median daily step count for each week, the variable of interest was postprocedure week, and we used a negative binomial distribution with log link. We included demographic variables (age, sex, health system) and procedure type (AF ablation or bariatric surgery). We used robust sandwich estimator for standard errors. The GEE method accounts for within-subject correlation of the observed outcomes and does not exclude an entire patient’s data if they are missing some observations.

#### Association between PROMs and step count

We compared median daily step counts between patients who did and those who did not report pain and palpitations using the Wilcoxon rank-sum test. We conducted a longitudinal analysis of the association of pain with median daily step count during the 5 weeks postprocedure with a GEE using the same methods described earlier. The variables of interest were (1) if a patient reported pain during a given week and (2) the interaction between the report of pain and the number of weeks postprocedure. We also used identical methods to examine the association of palpitations with median daily step count among patients who received AF ablation.

#### Association between mobile ECG recordings and step count

We compared median daily steps between patients with and those without recorded AF episodes using the Wilcoxon rank-sum test. We also conducted a longitudinal analysis of the association of number of AF recordings with median daily step count using a GEE, utilizing the same methods described for longitudinal analyses of the associations of pain and palpitations with median daily step count.

#### Association between PROMs and mobile ECG recordings

Among patients who performed at least 1 usable mobile ECG recording, we compared the median number of total recordings performed by patients with and those without recorded AF episodes using the Wilcoxon rank-sum test. To analyze the association between weeks with palpitations reported and AF or normal rhythm mobile ECG recordings, we constructed a GEE using exchange correlation structure. The dependent variable was report of palpitations, and we used a logistic regression with population-averaged estimator. The variable of interest was whether AF was detected, and we included demographic variables (age, sex, health system). We used robust sandwich estimators for standard errors.

#### Association of digital weight scale recordings with step count and PROMs

We analyzed the association between median daily steps and percentage body weight lost over 5 weeks using linear regression, controlling for age and sex. Procedure site (Yale or Mayo Clinic) was omitted due to collinearity. We also compared the percentage body weight lost by patients who did and those who did not report having an appetite during the 5-week follow-up period using the Wilcoxon rank-sum test.

#### Associations between clinical events (all patients) and antiarrhythmic medication prescriptions (AF ablation patients) and step count

We compared the median daily step count between patients who had an emergency department visit or hospitalization and those who did not using the Wilcoxon rank-sum test. We also compared the median number of total mobile ECG recordings performed in these 2 groups using the Wilcoxon rank-sum test. We also used the Wilcoxon rank-sum test to compare the median number of total and AF mobile ECG recordings performed by patients who received a new antiarrhythmic drug prescription and those who did not. These analyses included patients who performed no mobile ECG recordings. We used the Wilcoxon rank-sum test to compare the proportion of weeks with AF recording(s) between patients who received a new antiarrhythmic prescription and those who did not.

Statistical analyses were conducted using Excel Version 14.1.3 (Microsoft, Redmond, WA) and Stata Version 15.1 (StataCorp, College Station, TX).

## Results

### Study population characteristics

Of the 60 patients in the cohort study, 59 underwent their assigned procedure (30 ablation and 29 bariatric surgery). Twelve of 30 ablation patients (40%) and 22 of 29 bariatric surgery patients (76%) were female. Median [interquartile range] age of ablation patients was 64 [56–71] years and of bariatric surgery patients was 44 [39–57] years (Table 1).

**Table 1:** Patient cohort characteristics by procedure

	AF ablation patients	Bariatric surgery patients
Total	30	29
Sex		
Male	18 (60)	7 (24)
Female	12 (40)	22 (76)
Age (y)	64 [56–71]	44 [39–57]
Site		
Mayo Clinic	15 (50)	15 (52)
Yale–New Haven Hospital	15 (50)	14 (48)
Recorded steps∗		
Yes	21 (70)	26 (90)
No	9 (30)	3 (10)
Daily steps†	5132 [3131–7041]	3734 [2555–5746]
Reported pain		
Yes	17 (57)	19 (66)
No	12 (40)	9 (31)
Did not complete PROM	1 (3)	1 (3)
Reported palpitations		N/A
Yes	21 (70)	
No	8 (27)	
Did not complete PROM	1 (3)	
Reported appetite	N/A	
Yes		20 (69)
No		8 (28)
Did not complete PROM		1 (3)
Recorded AF episode		N/A
Yes	18 (60)	
No	7 (23)	
No usable recordings	5 (17)	
Emergency department or inpatient encounter		
Yes	3 (10)	4 (14)
No	27 (90)	25 (86)
New antiarrhythmic drug prescription		
Yes	3 (10)	N/A
No	27 (90)	N/A

Values are given as n, n (%), or median [interquartile range].

AF = atrial fibrillation; N/A = not applicable; PROM = patient-reported outcome measure.

∗ Reports whether or not the patient recorded at least 500 steps during at least 1 day over the 5-week follow-up period.

† Among patients who recorded steps.

### Step count assessment

Of the 59 patients, 47 (80%) recorded at least 500 steps during at least 1 day within the first 5 weeks of follow-up. Using data from these days, median daily step count was 4099 [2754–6853]; 5132 [3131–7041] among patients who underwent AF ablation and 3734 [2555–5746] among those who underwent bariatric surgery (Table 1). Median daily step count increased over time for both AF ablation and bariatric surgery patients (Supplemental Figures 1 and 2, respectively). When accounting for within-subject correlation of observations, each postprocedure week had a statistically significant marginal effect on median daily step count. Compared to week 1, postprocedure week 2 was associated with a median marginal increase of 1753 (95% confidence interval [CI] 969–2538; *P* <.01) steps; week 3 with 2079 (95% CI 1377–2782; *P* <.01) steps; week 4 with 2518 (95% CI 1694–3342; *P* <.01) steps; and week 5 with 2856 (95% CI 1995–3716; *P* <.01) steps.

### PROM assessment

Fifty-seven patients (97%) completed at least 1 pain PROM, 36 (63%) of whom reported pain at least once. Twenty-nine of the 30 ablation patients (97%) completed at least 1 palpitations PROM, 21 (72%) of whom reported palpitations at least once. Twenty-eight of the 29 bariatric surgery patients (97%) completed at least 1 appetite PROM, 20 (71%) of whom reported having an appetite at least once (Table 1).

**Figure 1:**
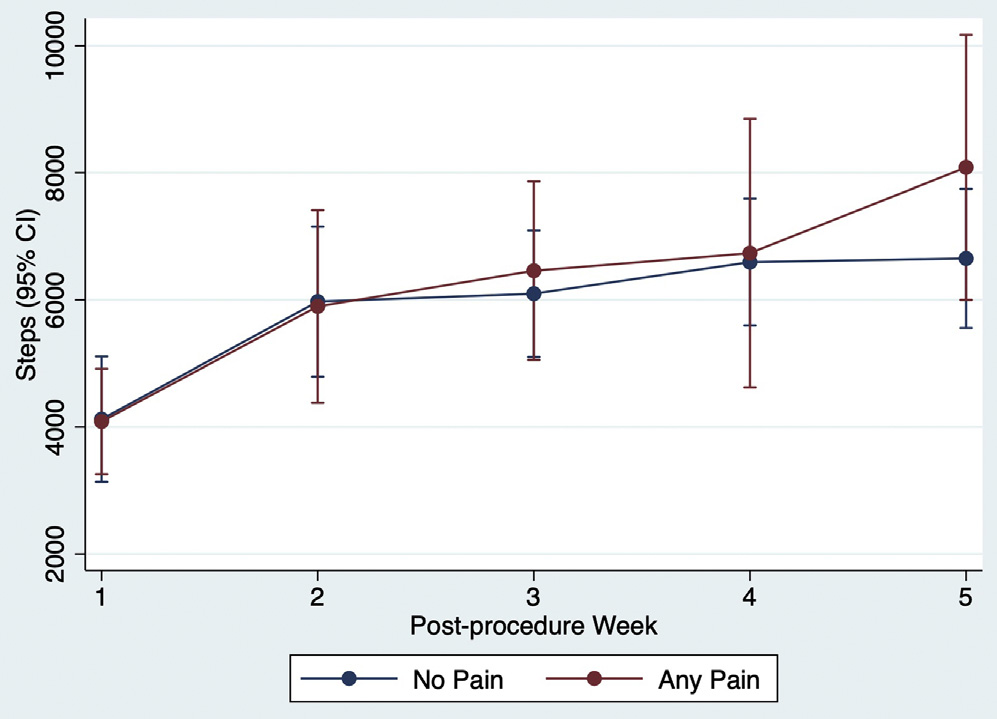
Average predicted median daily step count for patients during weeks with and without pain. CI = confidence interval.

There was no significant difference in median steps per day between patients who reported pain at least once (4419 [3286–7041]) and those who never reported pain (3498 [2609–5888]; *P* = .23) (Table 2). These results were consistent when limited to ablation patients only and to bariatric surgery patients only. When accounting for within-subject correlation of observations, reporting pain during the week did not have a significant marginal effect on median daily steps (374; 95% CI –490 to 1237; *P* = .40) compared to those not reporting pain during the week (Table 3). Figure 1 shows the change in median daily steps per week based on the presence or absence of reported pain.

**Table 2:** Median step count in patients with and without reported pain, reported palpitations, atrial fibrillation recordings, and emergency department or inpatient encounter

Outcome	Patients with outcome		Patients without outcome		*P* value
	N	Median steps [IQR]	N	Median steps [IQR]	
Pain†	30	4419 [3286–7041]	16	3498 [2609–5888]	.23
Palpitations	18	4668 [3021–6116]	3	8040 [6853–10,394]	.03∗
Atrial fibrillation‡	15	5132 [3286–7041]	6	4937 [3017–7198]	.88
Emergency department or inpatient encounter	5	4467 [1936–5421]	42	3811 [2754–6853]	.56

IQR = interquartile range.

∗ Statistically significant result, *P* <.05.

† Number of patients who reported pain includes both ablation patients and bariatric surgery patients, whereas the other outcomes only include patients who underwent ablation.

‡ Mobile electrocardiographic recordings detected as atrial fibrillation.

**Table 3:** GEE analysis of association of pain, palpitations, and AF episode with median daily step count

	Marginal effect on median daily step count (95% CI)		
	Independent variable: Pain	Independent variable: Palpitations	Independent variable: AF
Independent variable			
No	Reference	Reference	N/A
Yes	374 (–490 to 1237)	126 (–536 to 788)	–351 (–524 to –177)∗
Week			
1	Reference	Reference	Reference
2	1837 (936–2738)∗	1581 (901–2261)∗	1462 (346–2578)∗
3	2109 (1246–2971)∗	2290 (1352–3228)∗	2257 (1139–3375)∗
4	2532 (1445–3620)∗	2698 (1569–3827)∗	2622 (1411–3835)∗
5	3024 (1878–4170)∗	2756 (1465–4048)∗	2811 (1836–3786)∗
Sex			
Male	Reference	Reference	Reference
Female	–2381 (–4113 to –649)∗	–2569 (–5408 to 270)	–2690 (–4670 to –710)
Age†	2 (–84 to 88)	–3.2 (–116 to 110)	–7 (–91 to 77)
Site			
Mayo Clinic	Reference	Reference	Reference
Yale–New Haven Hospital	–1229 (–3271 to 812)	–1913 (–4853 to 1026)	–917 (–3067 to 1232)
Procedure			
Bariatric surgery	Reference	N/A	N/A
AF ablation	–1348 (–4204 – 1508)	N/A	N/A

AF = atrial fibrillation; CI = confidence interval; GEE = generalized estimating equation; N/A = not applicable.

∗ *P* <.05.

† Marginal steps per each additional year of age.

**Figure 2:**
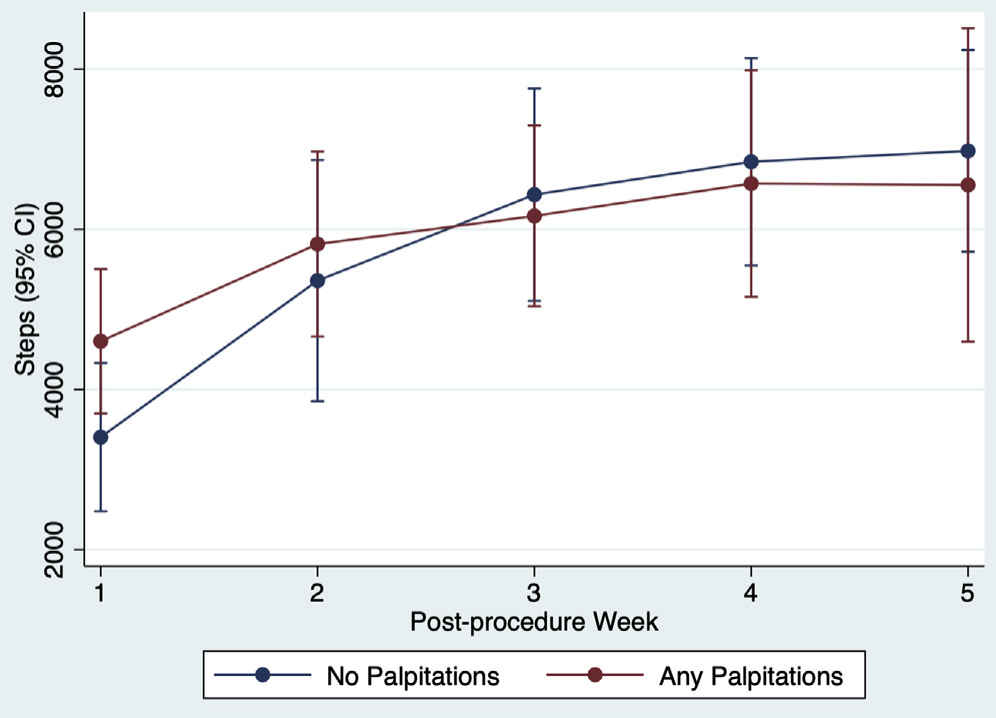
Average predicted median daily step count for weeks with and without palpitations. CI = confidence interval.

Median steps per day was significantly lower among ablation patients who reported palpitations at least once (4668 [3021–6116]) compared to those who never reported palpitations (8040 [6853–10,394]; *P* = .03) (Table 2). When accounting for within-subject correlation of observations, reporting palpitations during the week did not have a significant marginal effect on median daily steps (126; 95% CI –536 to 788; *P* = .71) compared to not reporting palpitations during the week (Table 3). Figure 2 shows the change in median daily steps per week based on the presence or absence of reported palpitations.

### Mobile ECG recording assessment (AF ablation patients)

Twenty-five ablation patients (83%) performed mobile ECG recordings at least once. A total of 891 recordings were performed, 702 (79%) of which were usable. Of the patients who performed recordings, the median number of usable recordings per patient was 20 [5–37].

Of the 25 patients with usable recordings, 18 (72%) had at least 1 episode of recorded AF. Median number of total mobile ECG recordings per patient with at least 1 AF recording was 39 [6–73] compared to 12 [4–41] among patients with no AF in any mobile ECG recording (*P* = .11) (Table 4). Among the 18 patients with at least 1 episode of AF, a median 26% [8%–50%] of these patients’ recordings showed AF.

**Table 4:** Median number of total mobile ECG recordings performed by all patients and by patients with and without at least 1 AF recording, an emergency department or inpatient encounter, and a new antiarrhythmic medication prescription (ablation patients only)

Independent variable	Patients with occurrence of independent variable		Patients without occurrence of independent variable		*P* value
	N	Median [IQR]	N	Median [IQR]	
All patients	30	16 [4–45]			
AF	18	39 [6–73]	7	12 [4–41]	.11
Emergency department or inpatient encounter	3	38 [0–40]	27	12 [4–45]	.76
New antiarrhythmic medication prescription	3	20 [0–45]	27	12 [4–45]	.81

AF = atrial fibrillation; ECG = electrocardiography; IQR = interquartile range.

**Figure 3:**
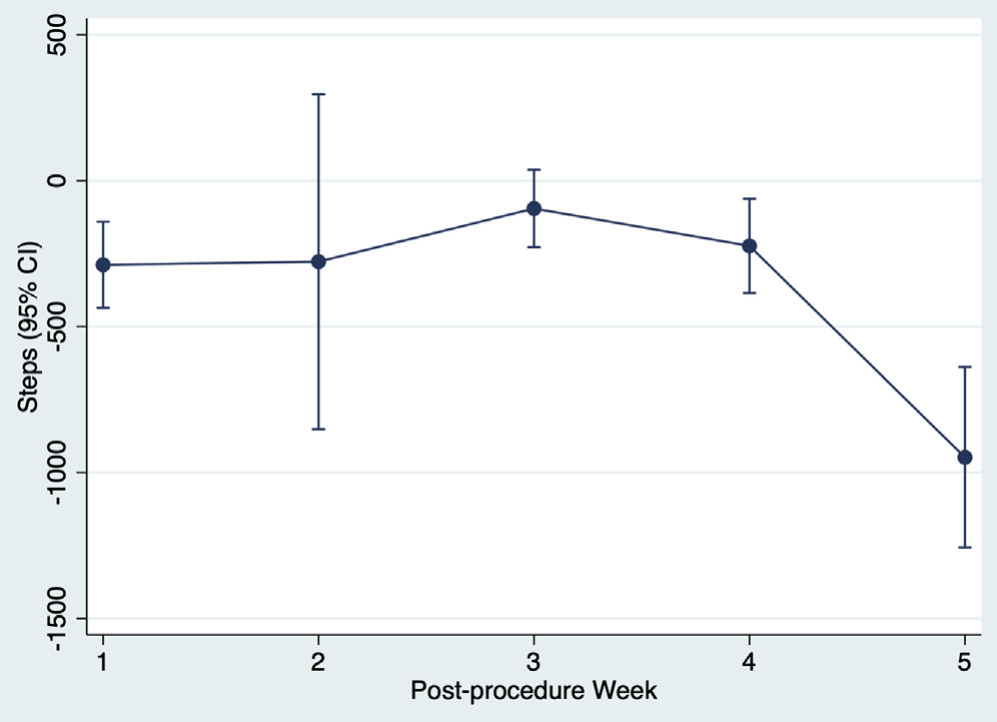
Marginal effect of an atrial fibrillation episode recorded on a mobile electrocardiographic device on median daily step count. CI = confidence interval.

Among patients who performed at least 1 usable mobile ECG recording, there was no difference in median overall steps per day among those who had at least 1 episode of AF recorded (5132 [3286–7041]; n = 15) and those who never had AF recorded (4937 [3017–7198]; n = 6) (*P* = .88) (Table 2). When accounting for within-subject correlation of observations, each AF episode during the week was associated with a significant mean marginal effect of –351 (95% CI –524 to –177; *P* <.01) median daily steps in that week (Table 3). An AF episode had a significantly negative mean marginal effect on median daily step count in postprocedure week 1 (–289; 95% CI –436 to –141; *P* <.01), week 4 (–224; 95% CI –385 to –62; *P* = .01), and week 5 (–948; 95% CI –1257 to –638; *P* <.01), and a nonsignificantly negative mean marginal effect on median daily step count in the other postprocedure weeks (Figure 3).

Twenty patients reported palpitations and performed at least 1 usable mobile ECG recording during 47 total weeks. In 23 of these 47 weeks (49%), the rhythm was AF, whereas in 24 (51%), the rhythm was sinus. The presence of an AF recording in a week was not significantly associated with a patient report of palpitations in the same week (odds ratio 2.60; 95% CI 0.85–7.96) (Supplemental Table 1).

### Digital weight scale recording assessment (bariatric surgery patients)

Twenty-two bariatric surgery patients (76%) performed a digital weight scale recording at least once. Of these patients, 11 (50%) recorded their weight in both weeks 1 and 5 postprocedure. Median percentage of weight lost during this time period was 6.1% [4.9%–7.8%].

Of the 11 patients who recorded their weight in both weeks 1 and 5, 10 (91%) also recorded at least 500 steps on at least 1 day during the postprocedure period. Each additional 1% weight lost between weeks 1 and 5 had a nonsignificant mean marginal effect of 658 (95% CI –1945 to 3263) steps (*P* = .56) (Supplemental Table 2). All patients who recorded their weight in both weeks 1 and 5 also completed at least 1 appetite PROM; 6 (55%) reported having an appetite at least once, and 5 (45%) did not report having an appetite. There was no difference in median percentage body weight lost in those who reported having an appetite (6.5% [4.9%–7.8%]) and those who did not (6.1% [5.3%–7.8%]) (*P* = .58).

### Clinical events (all patients) and new antiarrhythmic medication prescriptions (AF ablation patients)

Seven patients (12%) (3 ablation and 4 bariatric surgery) had an emergency department stay or hospitalization. Of the 30 ablation patients, 3 (10%) were prescribed a new antiarrhythmic medication more than 1 week after ablation. Two of these 3 patients (67%) recorded at least 1 AF episode every week before the prescription, whereas the other patient performed no mobile ECG recordings.

There was no statistically significant difference in steps per day between patients who had an emergency department or inpatient encounter (4467 [1936–5421]; n = 5) and those who did not (3811 [2754–6853]; n = 42) (*P* = .56) (Table 2).

There was no significant difference between the total number of mobile ECG recordings among patients who received a new antiarrhythmic medication prescription (20 [IQR 0–45]; n = 3) and those who did not (12 [4–45]; n = 27) (*P* = .81) (Table 4). There was a nonsignificantly higher median number of AF recordings performed by patients who received a new antiarrhythmic medication prescription (23 [16–29]; n = 2) compared to those who did not (2 [0–3]; n = 23) (*P* = .05). Median number of weeks with AF recording(s) was significantly higher for patients with a new antiarrhythmic prescription (5 [5–5]; n = 2 patients) than those without (1 [0–3]; n = 23 patients) (*P* = .02).

## Discussion

This study demonstrates that personal digital device data and PROMs can inform our understanding of postprocedural patient recovery after AF ablation and bariatric surgery. We did not find a significant difference in median daily step count between patients who reported pain or AF and those who did not. However, we did find a significant association between weeks with AF recordings and decreased median daily step count. This could indicate that comparisons utilizing granular patient-level data with intraindividual comparisons and trajectories may inform us about recovery better than broad comparisons among different patients.

Our study demonstrates that personal digital devices may provide information about the relationships between activity, symptoms, and clinical events. Previous studies showed that patients have lower activity measured by implantable cardioverter-defibrillators during AF episodes compared to before or after the episode.^25^ We found a similar association of AF episodes recorded by a mobile ECG device with lower step counts. We did not find an association between pain and physical activity, possibly because pain is unexpected after AF ablation.

Mobile ECG devices have been shown to detect AF recurrence after ablation or cardioversion earlier than standard of care but have not necessarily led to earlier treatment, presumably because data from these devices are not routinely integrated into clinical care.^14^ We found that patients who recorded at least 1 AF episode during each of the 5 weeks of follow-up were prescribed an antiarrhythmic medication. Similarly, a higher number of weeks with AF recordings and a nonsignificant increase in total AF episodes were recorded by patients who received an antiarrhythmic prescription compared to those who did not. Although the numbers are small, these results suggest that rhythm determination from personal digital devices may have utility in determining clinically meaningful AF recurrence immediately postablation and that these data could alert clinicians of events that require treatment.^14^ Integration of activity and symptom data with rhythm data from mobile ECG devices could inform earlier postablation rhythm control interventions, thereby potentially improving patient quality of life and possibly reducing the risk of long-term AF recurrence.^11^ Future studies should examine the potential for streaming personal digital device data, such as step counts and mobile ECG devices, to monitor patient recovery when paired with patient-reported outcomes, particularly when compared to preprocedural data. Tools that incorporate these multiple parameters may have significant potential to identify trajectories of recovery and detect possible complications before clinical manifestations.

It is important to ensure that patients use devices and share data frequently enough to aid clinical decision-making. Our previous analysis of this cohort study found that weekly Fitbit syncs fell from 47 patients (80%) in week 1 to 34 patients (58%) at week 8; 15 ablation patients (50%) performed mobile ECG recordings every week; and only 11 bariatric surgery patients (38%) recorded a weight every week.^21^ Interestingly, among patients receiving AF ablation, data from Fitbit were shared more often than KardiaMobile data, possibly because the Fitbit records steps automatically whereas KardiaMobile requires manual effort to perform a recording. We also found lower adherence to weight recordings among bariatric surgery patients than adherence to KardiaMobile recordings in AF patients. One possible reason is that AF symptoms could prompt patients to perform a KardiaMobile recording, compared to patients needing to remember to perform a weight recording. In the future, use of devices with automated functions may help improve adherence.

Additionally, device adherence may itself be an *indicator* of postprocedural recovery. Patients were instructed to use their mobile ECG device at least once weekly, and patients with AF recordings had a higher median number of total recordings. Although not significant, patients with postablation emergency department or inpatient visits and new antiarrhythmic medication prescriptions also had more recordings. These findings suggest that patients with AF or acute clinical conditions may be more likely to use their mobile ECG device to monitor their arrhythmia; both usage and content could provide early signals of potential clinical events. Another possibility is that patients may seem to have higher disease burden because they perform more recordings on their mobile ECG device (and therefore detect more AF episodes).

The increased use of telehealth and telemonitoring during the COVID-19 pandemic has increased the salience of personal digital device data in clinical decision-making, highlighting how these devices could inform postprocedural follow-up in the absence of in-person clinical encounters.^26^, ^27^, ^28^ For example, patients who are detected to be in sinus rhythm by a mobile ECG device, whose activity levels remain stable based on step count recorded by a personal digital device, and who report a lack of symptoms on PROMs may be able to avoid in-person follow-up after AF ablation. Similarly, the increasing emphasis on same-day discharge for procedures for which patients were generally hospitalized in the past could lead to the need for greater home monitoring through personal digital devices and PROMs.^29^ Digital health interventions have been shown to improve postdischarge follow-up and reduce health care costs.^30^ These devices may decrease patient burden while potentially improving outcomes through early detection of clinical deterioration. Although personal digital device and PROM data are not commonly a part of routine clinical care because of a lack of EHR integration, Medicare has augmented reimbursement for remote patient monitoring, and treatment paradigms may increase use.^31^, ^32^, ^33^ Future research analyzing larger samples and more granular personal digital device data may further elucidate meaningful associations with patient outcomes and guide clinical practice.

### Study limitations

First, although our results did not show a statistically significant difference in some outcome measures, our sample size was small and may have been underpowered. More data could better help to phenotype patients. Second, there were varying levels of data completeness, which is common to many real-world data studies, and methods are needed to handle missingness and appropriately impute data while supporting strategies to improve patient adherence. Third, we relied on mobile ECG recordings for diagnosis of AF instead of traditional sources such as 12-lead ECGs. However, these devices are increasingly being used for rhythm monitoring, and KardiaMobile has Food and Drug Administration 510(k) clearance to record ECGs and detect normal sinus rhythm and AF in adults.^34^

## Conclusion

We collected data from personal digital devices, PROMs, and EHRs for 5 weeks after AF ablation and bariatric surgery to analyze associations between activity, symptoms, and clinical events. Although we did not find significant differences in activity between patients based on some symptom and personal digital device data, we found an inverse association between AF episodes recorded and activity. Our results demonstrate that the associations between personal digital device, PROM, EHR, and pharmacy data may provide insight into postprocedural recovery and inform follow-up and clinical decision-making.

## Acknowledgments

We gratefully acknowledge the National Evaluation System for Health Technology (NEST) designation of this work as a Demonstration Project and the Hugo team for their assistance throughout this project. We acknowledge AliveCor for their generous donation of the KardiaMobile devices used in this study. Most importantly, we gratefully acknowledge the contributions of patients to this study.

## Funding Sources

This work was supported in part by a Center of Excellence in Regulatory Science and Innovation (CERSI) grant to Yale University and Mayo Clinic from the US Food and Drug Administration (FDA) (U01FD005938) and by Johnson & Johnson. Its contents are solely the responsibility of the authors and do not necessarily represent the official views nor the endorsements of the Department of Health and Human Services, FDA, or Johnson & Johnson.

## Disclosures

Dr Dhruva currently receives research support from the National Heart, Lung, and Blood Institute (NHLBI) of the National Institutes of Health (NIH) (K12HL138046); Food and Drug Administration (FDA); National Evaluation System for Health Technology (NEST) Coordinating Center; Arnold Ventures; and the Greenwall Foundation. Dr Noseworthy receives research funding from the NIH, including the NHLBI and the National Institute on Aging (NIA), Agency for Healthcare Research and Quality (AHRQ), FDA, and American Heart Association (AHA); and is a study investigator in an ablation trial sponsored by Medtronic. Dr Noseworthy and Mayo Clinic are involved in potential equity/royalty relationship with AliveCor. Dr Noseworthy has served on an expert advisory panel for Optum. Dr Noseworthy and Mayo Clinic have filed patents related to the application of artificial intelligence to the ECG for diagnosis and risk stratification. Dr Ross formerly received research support through Yale University from Medtronic, Inc., and the FDA to develop methods for postmarket surveillance of medical devices (U01FD004585); from the Centers of Medicare and Medicaid Services (CMS) to develop and maintain performance measures that are used for public reporting (HHSM-500-2013-13018I); and from the Blue Cross Blue Shield Association to better understand medical technology evaluation; and currently receives research support through Yale University from Johnson & Johnson to develop methods of clinical trial data sharing; the Medical Device Innovation Consortium as part of NEST; AHRQ (R01HS022882); NHLBI of the NIH (R01HS025164); and the Laura and John Arnold Foundation to establish the Good Pharma Scorecard at Bioethics International and to establish the Collaboration for Research Integrity and Transparency (CRIT) at Yale. In the past 36 months, Dr Shah has received research funding from the Centers of Medicare and Medicaid Innovation under the Transforming Clinical Practice Initiative (TCPI); AHRQ (R01HS025164, R01HS025402, 1U19HS024075, R03HS025517); NHLBI of the NIH (R56HL130496, R01HL131535); National Science Foundation; and Patient-Centered Outcomes Research Institute (PCORI) to develop a Clinical Data Research Network (LHSNet).

## Data Availability

The dataset generated and analyzed for this study will not be made publicly available due to patient privacy and lack of informed consent to allow sharing of patient data outside of the research team.

## Authorship

All authors attest they meet the current ICMJE criteria for authorship.

## Patient Consent

All patients provided written informed consent.

## Ethics Statement

The authors designed the study, and gathered and analyzed the data according to the Helsinki Declaration as revised in 2013. The research protocol used in this study was reviewed and approved by institutional review boards at Yale University and Mayo Clinic.

## References

[1] Cook D.J., Thompson J.E., Prinsen S.K., Dearani J.A., Deschamps C. (2013). Functional recovery in the elderly after major surgery: assessment of mobility recovery using wireless technology. Ann Thorac Surg.

[2] Twiggs J., Salmon L., Kolos E., Bogue E., Miles B., Roe J. (2018). Measurement of physical activity in the pre- and early post-operative period after total knee arthroplasty for osteoarthritis using a Fitbit Flex device. Med Eng Phys.

[3] McClung H.L., Ptomey L.T., Shook R.P. (2018). Dietary intake and physical activity assessment: current tools, techniques, and technologies for use in adult populations. Am J Prev Med.

[4] Gilmore S.J., Hahne A.J., Davidson M., McClelland J.A. (2020). Physical activity patterns of patients immediately after lumbar surgery. Disabil Rehabil.

[5] Dhruva SS, Ross JS, Shah N, Fleurence R, Krumholz HM. New Federal Rules Pave the Way for Patient-Driven Health Information Exchange and Real-World Evidence on COVID-19 Surveillance and Treatment. May 10, 2020. Health Affairs Blog. https://doi.org/10.1377/hblog20200506.368396.

[6] Craig T.J., Wann S.L., Calkins H. (2019). 2019 AHA/ACC/HRS focused update of the 2014 AHA/ACC/HRS guideline for the management of patients with atrial fibrillation: a report of the American College of Cardiology/American Heart Association Task Force on Clinical Practice Guidelines and the Heart Rhythm Society in Collaboration With the Society of Thoracic Surgeons. Circulation.

[7] Kannel W.B., Abbott R.D., Savage D.D., McNamara P.M. (1982). Epidemiologic features of chronic atrial fibrillation: the Framingham study. N Engl J Med.

[8] Calkins H., Kuck K.H., Cappato R. (2012). 2012 HRS/EHRA/ECAS expert consensus statement on catheter and surgical ablation of atrial fibrillation: recommendations for patient selection, procedural techniques, patient management and follow-up, definitions, endpoints, and research trial design: a report of the Heart Rhythm Society (HRS) Task Force on Catheter and Surgical Ablation of Atrial Fibrillation. Developed in partnership with the European Heart Rhythm Association (EHRA), a registered branch of the European Society of Cardiology (ESC) and the European Cardiac Arrhythmia Society (ECAS); and in collaboration with the American College of Cardiology (ACC), American Heart Association (AHA), the Asia Pacific Heart Rhythm Society (APHRS), and the Society of Thoracic Surgeons (STS). Endorsed by the governing bodies of the American College of Cardiology Foundation, the American Heart Association, the European Cardiac Arrhythmia Society, the European Heart Rhythm Association, the Society of Thoracic Surgeons, the Asia Pacific Heart Rhythm Society, and the Heart Rhythm Society. Heart Rhythm.

[9] Berkowitsch A., Greiss H., Vukajlovic D. (2005). Usefulness of atrial fibrillation burden as a predictor for success of pulmonary vein isolation. Pacing Clin Electrophysiol.

[10] Kim Y.G., Boo K.Y., Choi J.-I. (2021). Early recurrence is reliable predictor of late recurrence after radiofrequency catheter ablation of atrial fibrillation. JACC Clin Electrophysiol.

[11] Liu J., Yang H., Liu Y. (2020). Early recurrence of atrial tachyarrhythmia during the 90-day blanking period after cryoballoon ablation in patients with atrial fibrillation: the characteristics and predictive value of early recurrence on long-term outcomes. J Electrocardiol.

[12] Gościniak P., Kowalik I., Sielicki P., Brykczyński M. (2013). Predictors of short- and mid-term recurrence of atrial fibrillation after surgical radiofrequency ablation: a six-month transtelephonic electrocardiogram monitoring study. Cardiol J.

[13] Wechselberger S., Kronborg M., Huo Y. (2018). Continuous monitoring after atrial fibrillation ablation: the LINQ AF study. Europace.

[14] Goldenthal I.L., Sciacca R.R., Riga T. (2019). Recurrent atrial fibrillation/flutter detection after ablation or cardioversion using the AliveCor KardiaMobile device: iHEART results. J Cardiovasc Electrophysiol.

[15] Athyros V.G., Tziomalos K., Karagiannis A., Mikhailidis D.P. (2011). Cardiovascular benefits of bariatric surgery in morbidly obese patients. Obes Rev.

[16] Herring L.Y., Stevinson C., Davies M.J. (2016). Changes in physical activity behaviour and physical function after bariatric surgery: a systematic review and meta-analysis. Obes Rev.

[17] Possmark S., Sellberg F., Willmer M., Tynelius P., Persson M., Berglind D. (2020). Accelerometer-measured versus self-reported physical activity levels in women before and up to 48 months after Roux-en-Y Gastric Bypass. BMC Surg.

[18] Nielsen M.S., Alsaoodi H., Hjorth M.F., Sjödin A. (2021). Physical activity, sedentary behavior, and sleep before and after bariatric surgery and associations with weight loss outcome. Obes Surg.

[19] Nikolić M., Kruljac I., Kirigin L. (2015). Initial weight loss after restrictive bariatric procedures may predict mid-term weight maintenance: results from a 12-month pilot trial. Bariatr Surg Pract Patient Care.

[20] Mor A., Sharp L., Portenier D., Sudan R., Torquati A. (2012). Weight loss at first postoperative visit predicts long-term outcome of Roux-en-Y gastric bypass using Duke weight loss surgery chart. Surg Obes Rel Dis.

[21] Dhruva S.S., Ross J.S., Akar J.G. (2020). Aggregating multiple real-world data sources using a patient-centered health-data-sharing platform. NPJ Digit Med.

[22] Block V.J., Lizée A., Crabtree-Hartman E. (2017). Continuous daily assessment of multiple sclerosis disability using remote step count monitoring. J Neurol.

[23] Semaan S., Dewland T.A., Tison G.H. (2020). Physical activity and atrial fibrillation: data from wearable fitness trackers. Heart Rhythm.

[24] Chen W., Liu H., Ling Z. (2016). Efficacy of short-term antiarrhythmic drugs use after catheter ablation of atrial fibrillation—a systematic review with meta-analyses and trial sequential analyses of randomized controlled trials. PLoS One.

[25] Chelu M.G., Gunderson B.D., Koehler J., Ziegler P.D., Sears S.F. (2016). Patient activity decreases and mortality increases after the onset of persistent atrial fibrillation in patients with implantable cardioverter-defibrillators. JACC Clin Electrophysiol.

[26] Mehrotra A., Chernew M., Linetsky D., Hatch H., Cutler D., Schneider E.C. The Impact of the COVID-19 Pandemic on Outpatient Visits: Changing Patterns of Care in the Newest COVID-19 Hot Spots. August 13, 2020. https://www.commonwealthfund.org/publications/2020/aug/impact-covid-19-pandemic-outpatient-visits-changing-patterns-care-newest.

[27] Varma N., Marrouche N.F., Aguinaga L. (2020). HRS/EHRA/APHRS/LAHRS/ACC/AHA worldwide practice update for telehealth and arrhythmia monitoring during and after a pandemic. Circ Arrhythm Electrophysiol.

[28] Varma N., Cygankiewicz I., Turakhia M.P. (2021). 2021 ISHNE/HRS/EHRA/APHRS expert collaborative statement on mHealth in arrhythmia management: digital medical tools for heart rhythm professionals: from the International Society for Holter and Noninvasive Electrocardiology/Heart Rhythm Society/European Heart Rhythm Association/Asia-Pacific Heart Rhythm Society. Circ Arrhythm Electrophysiol.

[29] Deyell M.W., Leather R.A., Macle L. (2020). Efficacy and safety of same-day discharge for atrial fibrillation ablation. JACC Clin Electrophysiol.

[30] Cittanova M.-L., Chauvier S., Combettes E. (2021). Association of automated text messaging with patient response rate after same-day surgery. JAMA Netw Open.

[31] Mori M., Jarrin R., Krumholz H.M. Ambiguities in New Schedules for Reimbursing Digital Medical Services in the US Must Be Clarified. November 20, 2020. BMJ Opinion. https://blogs.bmj.com/bmj/2020/11/20/ambiguities-in-new-schedules-for-reimbursing-digital-medical-services-in-the-us-must-be-clarified/.

[32] Dey P., Jarrin R., Mori M., Geirsson A., Krumholz H.M. (2021). Leveraging remote physiologic monitoring in the COVID-19 pandemic to improve care after cardiovascular hospitalizations. Circ Cardiovasc Qual Outcomes.

[33] Mecklai K., Smith N., Stern A.D., Kramer D.B. (2021). Remote patient monitoring—overdue or overused?. N Engl J Med.

[34] US Food and Drug Administration. KardiaMobile, KardiaStation 510(k). 2020. https://www.accessdata.fda.gov/cdrh_docs/pdf19/K191406.pdf. Accessed July 20, 2021.

