# Supplementary material for: Physical activity, patient-reported symptoms, and clinical events: Insights into postprocedural recovery from personal digital devices
Source: Cardiovasc Digit Health J. 2021 Jul 3;2(4):212–21. doi: 10.1016/j.cvdhj.2021.06.002 (PMC8890038; doi:10.1016/j.cvdhj.2021.06.002)
Supplement: Supplemental Tables 1, 2 and Supplemental Figures 1, 2 [file mmc1.docx]

**Supplemental Table 1**: Generalized Estimating Equation Analysis of Association of Atrial Fibrillation Episodes with Palpitations

| **Variable** | **Odds Ratio (95% CI) of Reporting Palpitations** |
| --- | --- |
| AF episode(s) recorded |  |
| No | Reference |
| Yes | 2.60 (0.85–7.96) |
| Week |  |
| 1 | Reference |
| 2 | 1.04 (0.27–3.93) |
| 3 | 0.55 (0.18–1.73) |
| 4 | 0.69 (0.18–2.57) |
| 5 | 0.40 (0.12–1.38) |
| Sex |  |
| Male | Reference |
| Female | 1.26 (0.36–4.39) |
| Age† | 0.93 (0.88–0.98)* |
| Site |  |
| Mayo Clinic | Reference |
| Yale–New Haven Hospital | 0.94 (0.28–3.15) |

**P* <.05.

†Marginal steps per each additional year of age.

**Supplemental Table 2:** Linear Regression Analysis of Percentage Weight Lost and Median Daily Step Count

| **Variable** | **Marginal Effect on Median Daily Step Count (95% CI)** |
| --- | --- |
| Percent weight lost | 658 (–1945 to 3263) |
| Sex |  |
| Male | Reference |
| Female | 1164 (–7962 to 10,290) |
| Age* | 0.55 (–337 to 339) |

Note: Procedure site (Yale–New Haven Hospital or Mayo Clinic) omitted because of collinearity.

*Marginal steps per each additional year of age.

**Supplementary Figure 1:** Median Daily Steps by Postprocedure Day for Atrial Fibrillation Ablation Patients

**Supplementary Figure 2:** Median Daily Steps by Postprocedure Day for Bariatric Surgery Patients
